# Plasmonic Nanosensors for EGFR Detection: Optimizing Aptamer-Based Competitive Displacement Assays

**DOI:** 10.3390/bios15100699

**Published:** 2025-10-15

**Authors:** Alexandra Falamas, Andra-Sorina Tatar, Sanda Boca, Cosmin Farcău

**Affiliations:** 1 National Institute for Research and Development of Isotopic and Molecular Technologies, 67-103 Donat, 400293 Cluj-Napoca, Romania; alexandra.falamas@itim-cj.ro (A.F.); andra.tatar@itim-cj.ro (A.-S.T.); sanda.boca@ubbcluj.ro (S.B.); 2 Interdisciplinary Research Institute in Bio-Nano-Sciences, Babes-Bolyai University, 42 Treboniu Laurian, 400271 Cluj-Napoca, Romania

**Keywords:** biosensor, nanosensing, optical sensor, molecular diagnostics, surface plasmon resonance, fluorescence, AuFoN, analytical chemistry

## Abstract

This study presents a comparative investigation of plasmonic sensing platforms based on colloidal gold nanoparticle (AuNP) suspensions and gold film over nanosphere (AuFoN) solid substrates for the detection of epidermal growth factor receptor (EGFR), an essential biomarker and therapeutic target in oncology. The strategy relies on fluorescence emission modulation of an Atto647N-labeled DNA oligomer competitively bound to an EGFR-specific aptamer. Our results demonstrate that the colloidal AuNPs can function as competitive binding sensors, leading to fluorescence quenching upon fluorophore attachment to the surface of the NPs and partial fluorescence recovery due to EGFR-induced displacement of the fluorophore–aptamer complex. This specificity was confirmed by reversed binding experiments. However, the system proved highly sensitive to the experimental design: excessive washing (centrifugation) led to unspecific aggregation and signal loss, while reduced washing steps improved signal retention and revealed EGFR-induced fluorophore displacement into the supernatant. On the contrary, film-based substrates exhibited strong initial fluorescence, but failed to retain the fluorophore–aptamer complex after washing, resulting in fluorescence decay independent of EGFR incubation. This indicates that AuFoN lacked the binding stability necessary for specific displacement-based sensing. These findings highlight that while colloidal AuNPs can support competitive binding detection, their reproducibility is limited by colloidal stability and protocol sensitivity, whereas AuFoN substrates require improved surface functionalization strategies. The study emphasizes the critical role of surface chemistry, aptamer–fluorophore affinity, and washing protocols in determining the success or failure of plasmon-enhanced aptamer-based biosensing systems and suggests opportunities for improving specificity and robustness in future designs.

## 1. Introduction

Recent advances in aptamer technology have established these ligands as versatile tools for precise molecular recognition [1]. Aptamers are short, single-stranded nucleic acid sequences (oligonucleotide) obtained through a process known as systematic evolution of ligands by exponential enrichment (SELEX) [2,3]. SELEX is an iterative in vitro selection method used to identify single-stranded nucleic acid sequences that bind with high affinity and specificity to a given target. The resulting aptamers often display a nanomolar to picomolar dissociation constant K_d_, which evaluates the affinity of the aptamer for the target molecule. They offer a compelling alternative to antibodies due to their ease of synthesis, chemical stability, and minimal immunogenicity [4,5]. Aptamers, after having their optimal sequence identified by SELEX, can be obtained through chemical, solid-phase phosphoramidite synthesis, using automated DNA synthesizers [6]. Moreover, proteins are more sensitive to freeze-thaw cycles and degradation compared to deoxyribonucleic acids. By integrating aptamers onto gold nanostructured surfaces, complex biosensing assemblies can be constructed that respond to specific interactions [7]. Such aptasensors have been explored for the detection of clinically relevant biomarkers [8,9], environmental toxins [10], pathogens [11,12], and various other types of targets [13]. These systems typically convert the molecular recognition event into a measurable signal.

Optical detection methods, in particular, have gained considerable interest due to their sensitivity, real-time readout capabilities, and compatibility with miniaturized and multiplexed formats. Plasmonic nanostructures exhibit unique light–matter interactions due to the emergence of localized surface plasmon resonance (LSPR)—the coherent oscillations of conduction electrons induced by incident light. Such platforms exploit the LSPR to enable highly sensitive detection of molecular interactions [14]. While LSPR wavelength shifts due to colloidal particle aggregation or by adsorption of molecules with different refractive indices enable label-free detection [15,16], the proximity of molecules to the metallic nanostructures can additionally alter their intrinsic optical properties, such as Raman scattering or fluorescence emission.

Surface-enhanced fluorescence (SEF), also known as metal-enhanced fluorescence (MEF), occurs when plasmon-driven excitation enhancement and an increased radiative decay rate overcome non-radiative losses, leading to higher brightness and shorter lifetimes. In contrast, SERS depends on the fourth power of the local field magnitude, |E|^4^, and is dominated by nanoscale “hotspots” where the field enhancement is maximal, producing very large Raman cross-sections even under conditions where fluorescence is quenched.

Plasmonic nanostructures amplify the local electric field, thereby increasing the excitation rate of nearby molecules, and simultaneously alter the emitter’s total decay rate (Г_tot_ = Г_rad_ + Г_nr_ + Г_metal_). The metal can either enhance the radiative decay rate, Г_rad_, or introduce an additional non-radiative channel, Г_metal_, that leads to fluorescence quenching. Net fluorescence enhancement therefore occurs only when the combined increase in excitation and radiative coupling outweigh the non-radiative losses [17]. This effect is strongly distance-dependent, with maximal enhancement typically observed at separations of a few nanometres and quenching dominating at very short distances from the metal surface [18]. Furthermore, the geometry of metal nanostructures, the spectral overlap between plasmon resonance and fluorophore absorption/emission, and the molecular orientation all regulate the extent and direction of the fluorescence modulation.

In SERS, the electromagnetic enhancement is extremely sensitive to high field hotspots, such as gaps or junctions. Thus, even if a molecule is close enough to the metal surface for its fluorescence to be quenched, the Raman effect still benefits from the strongly amplified field, since Raman scattering is an instantaneous inelastic process [19]. SERS applications, both label-based and label-free configurations [20,21,22], have been shown to offer exceptional, several orders of magnitude enhancement factors [23], whilst SEF yields signal modulation through quenching or enhancement mechanisms dependent on the distance between the metal surface and the dye molecule [24,25,26]. Therefore, countless configurations of ON/OFF fluorescence-based aptasensors may be designed, with small molecular interactions such as recognition events or complementary strand binding leading to signal-switching effects that enable real-time detection with minimal background interference.

To transduce analyte binding into measurable optical response, various types of plasmonic nanostructures, including colloidal nanoparticles or solid nanostructured surfaces, can be employed. Gold film over nanosphere (AuFoN) substrates are robust and uniform substrates, offering large surface area and tunable optical properties by modifying the diameter of the nanospheres [27]. They are widely used, especially in SERS biosensing applications [28], offering high enhancement factors; however, fewer fluorescence-based biosensing reports can be found in the scientific literature. Still, fluorescence-based sensing using AuFoN remains relatively underexplored and presents practical challenges related to photophysical interactions and biomolecular stability.

An essential rationale for employing an aptamer-based detection strategy for applications using the SEF effect is the possibility to control the distances between the dye molecules and the metal surface. As such, the physical size of an antibody can bring the target to a distance of 11–14 nm [29] from the metal surface. All the while, each nucleotide has approximately 0.34 nanometers (3.4 Angstrom), meaning that the varying length of an aptamer makes the metal–molecule distance tunable based on the aptamer sequence, in combination with potential additional spacer nucleotides. Fluorescence-based aptasensing employing ON/OFF mechanisms have been reported for various biomarkers and fluorophores. Ag_2_S quantum dots were employed to quench the emission of the aptamer/5–fluoroacil complex, while biomarker detection resulted in specific binding to the complex, which led to its separation from the quantum dot and photoluminescence recovery [30]. Detection limits of prostate specific antigen down to the picomolar range were obtained through enzymatic cleavage of its corresponding aptamer using AuNPs and fluorescein isothiocyanate dye. Moreover, controlled MEF was obtained by keeping the nanoparticles and fluorophore molecules at a fixed distance of 7 nm using ssDNA and a short peptide [31]. Through the increased localized electromagnetic fields, nanostructured plasmonic substrates offer enhanced fluorescence emission and sensitive detection, as well as the ability to multiplex and detect more biomarkers in one step [32]. For example, silver-coated ZnO gratings enabled EGFR detection with 300× fluorescence enhancement and a detection limit down to the hundreds of femtomolar range [33].

A viable alternative is offered by electrochemical (EC)-based aptamer assays and sensors including enzyme-based assays, electrochemical impedance spectroscopy, and sensors using nanomaterials. Although some electrochemical aptasensors have shown high sensitivity, most of them imply costly and complicated fabrication steps, intricate modification of aptamers, and functionalization of nanomaterials [34,35]. EC sensors work by directly transducing the aptamer–target binding into an electrical signal. They typically offer high sensitivity and low detection limits [36], and are miniaturized into portable devices, enabling point-of-care testing [37]. However, electrochemical aptamer sensors are often less sensitive, harder to multiplex, and more prone to environmental interference than fluorescence-based sensors. Fluorescence-based aptasensors, while more prone to batch-to-batch sensitivity and reproducibility challenges, provide unique advantages such as real-time monitoring of aptamer–target binding, optical readout compatible with microscopy and imaging platforms, the possibility of multiplexing using spectrally different fluorophores, and integration with plasmonic nanostructures to achieve strong signal enhancement. These aspects make them attractive for exploiting plasmon–fluorophore interactions and competitive binding strategies for ON-OFF optical signals.

One of the least addressed but most critical issues in aptamer-based plasmonic sensing is the reproducibility of the fluorescence response, which is often compromised by subtle experimental parameters: the role of surface chemistry and post-functionalization handling, especially washing and centrifugation steps. Washing is necessary to remove excess ligands, but it also introduces mechanical and chemical stress that can promote nanoparticle aggregation, detach loosely bound molecules, or disrupt the aptamer’s secondary structure. Despite the wide use of these steps in biosensor protocols, their impact on signal retention, specificity, and reproducibility is rarely quantified in detail. Equally important, fluorescence quenching may occur not only via proximity-induced plasmonic interactions, but also through dye aggregation, improper folding of the aptamer, or nonspecific adsorption of the fluorophore. Restoration of the signal upon displacement is further complicated by steric hindrance, weak binding of the reporter strand, or irreversible surface adsorption. These effects introduce variability that may obscure the detection of specific binding events.

A particularly relevant target for aptamer-based diagnostics is the epidermal growth factor receptor (EGFR), a pivotal biomarker and therapeutic target in oncology with substantial implications in tumour progression and prognosis. EGFR plays a central role in modulating key cellular characteristics, such as cell morphology, differentiation, proliferation, and apoptosis, under normal physiological conditions. Aberrations in the regulatory mechanisms, such as increased expression or hyperactivation of EGFR, are frequently linked to the development of malignancies. In response, recent research has been focused on several EGFR-targeted therapies, including monoclonal antibodies (mAbs) [38], small molecule tyrosine kinase inhibitors (TKIs) [39], and aptamers. The latter has emerged as a promising alternative to mAbs: aptamer synthesis is simpler, more straightforward, and much more cost-effective compared to the production of antibodies, which require genetic knowledge about the specific antibody, cell cultures, and/or animal sacrifice. To date, the literature concerning fluorescence-based plasmonic aptasensing of EGFR is scarce.

Herein, we explore the interaction dynamics between the EGFR protein, an EGFR-specific aptamer, and a novel complementary fluorophore-labelled DNA strand, assembled either on suspension-based colloidal gold nanoparticles or on solid substrates consisting of gold film over nanospheres. Using a combination of fluorescence spectroscopy, quantum yield measurements, and UV–Vis extinction or reflectance profiling, we investigated how functionalization steps, washing procedures, and substrate type affect the photophysical response of the system. Our goal is to identify critical parameters that affect biosensor fidelity and to provide improved design guidelines for more robust and reproducible next-generation aptamer-based plasmonic biosensor platforms with enhanced stability and operational consistency. Our results show that the reliable development of such plasmonic nanosensors is highly sensitive to multiple parameters, including colloidal stability, which would ultimately influence the degree of nanoparticle aggregation, and batch-to-batch variation of nanoparticle–conjugate complexes. In particular, the application of multiple washing steps led to substantial loss of fluorescence, and different AuNPs batches resulted in inconsistent sensing performance. On solid substrates, fluorescence signal degradation and non-specific release of the fluorophore further complicated detection. These unforeseen or less favourable outcomes are instrumental in illuminating the inherent constraints and areas for improvement of aptamer-based plasmonic biosensing platforms. Our findings should be seen as providing mechanistic insight into the interplay between variations among batches of Au colloidal NPs, surface chemistry, and fluorophore stability in plasmon-enhanced aptasensing, which are critical for future biosensor design.

## 2. Materials and Methods

### 2.1. Materials

Hydrogen tetrachloroaurate (III) trihydrate (HAuCl_4_·3H_2_O, 99.99%) and trisodium citrate (Na_3_C_6_H_5_O_7_) were obtained from Sigma-Aldrich. The oligomers were ordered from Kaneka Eurogentec S.A. (Belgium), as follows: aptamer “M” sequence GGA-CGG-ATT-TAA-TCG-CCG-TAG-AAA-AGC-ATG-TCA-AAG-CCG-GAA-CCG-TCC with a 3′-Thiol modifier, and oligomer GCT-TTT-CTA-CGG-C with a 5′-ATTO 647N modification, named “Atto” throughout the article. Tris-EDTA buffer was acquired from AppliChem GmbH. All other reagents used during the experiments were of analytical grade and were utilized without further purification. Double-distilled water was used for the preparation of solutions and rinsing of the glassware.

### 2.2. Colloidal Nanoparticles: Synthesis, Functionalization, and Competitivity Assay

Colloidal gold nanoparticles were synthesized by the aqueous reduction of HAuCl_4_ with trisodium citrate in a variation of the Turkevich–Frens method [40]. Briefly, 50 mL of 0.25 × 10^−3^ M HAuCl_4_ × 3 H_2_O was boiled under stirring, then a solution of 38.8 × 10^−3^ M sodium citrate was quickly added. The formation of colloidal nanoparticles was indicated by the change of the solution colour from yellow to pinkish-red. At this point, the heating was stopped, while the stirring process continued for another 10–15 min.

In order to ensure full aptamer surface coverage, we incubated an excess of aptamers. Our calculations, based on the Sigma-Aldrich technical data on AuNPs [41], showed a molar absorption coefficient or molar attenuation coefficient (ε) of 1.72 × 10^10^ for 50 nm AuNPs, corresponding to a 535 nm LSPR. Thus, for a colloid of 0.16 absorption, we calculated a molar concentration of the particles of 4.65 × 10^11^, or 2.8 × 10^10^ particles per milliliter, with a total surface area of 2.2 × 10^14^ nm^2^ per milliliter. Considering an aptamer footprint of 12 nm^2^ for the coverage of a 50 nm diameter sphere [42], total surface coverage of 1 mL of our AuNPs would be accomplished by 1.83 × 10^13^ thiolated oligomers, which would translate to 0.3 μL. A four-fold excess of aptamer M was used for incubation to ensure full AuNP surface coverage, and a subsequent washing step cleared the excess unbound molecules. For purification by centrifugation, we settled on 6000 rcf for 10 min, in order to ensure good deposition of the particles and a clear supernatant, without the formation of gold pellets that disturb colloidal stability. Purified particles were resuspended in water.

For the identification of an optimal configuration of the developed nanosensor, we explored the binding affinity between three molecular components: the EGFR-specific M aptamer as the main binding element, a 13-nucleotide complementary Atto647N oligomer as the fluorescent tag, and the EGFR protein. Precisely, a two-pronged approach was employed: (i) linking Atto to the M-functionalized AuNPs (AuNPs_M_Atto) followed by the addition of EGFR, and (ii) incubating EGFR with the M-functionalized AuNPs (AuNPs_M_EGFR) followed by addition of Atto. The fluorescence of Atto was tracked throughout the experimental steps and used as an indicator for the binding affinities of EGFR vs. Atto toward the M aptamer. The final concentration of M and Atto was 1.2 × 10^−7^ M (1.2 μL of 10^−4^ M for every 1 mL of AuNPs), and EGFR was added in a final concentration of 8.8 × 10^−10^ M (3 μL of 3 × 10^−7^ M for every 1 mL of AuNPs). For a clearer image of the results, the biosensing protocol/competitivity assay is systematized in Table 1.

### 2.3. AuFoN Substrate Fabrication

The fabrication of the gold film over nanosphere substrates was elaborated previously [43]. Briefly, colloidal nanosphere monolayer arrays were obtained by convective self-assembly of polystyrene (PS) nanospheres (500 nm diameter) on PS plates. A 120 nm thick gold film was deposited by e-beam evaporation (Kenosistec KE400) on top of the PS nanospheres.

### 2.4. Measurement Techniques: Reflectivity and Fluorescence

The optical absorbance spectra of gold nanoparticles and of the oligomer-gold nanoparticles in aqueous solution were obtained with a Jasco V-750 UV−Vis−NIR spectrophotometer (Jasco Inc.,Tokyo, Japan). The dimensions of the as-prepared nanoparticles were determined by transmission electron microscopy (TEM) measurements using a JEOL model JEM 1010 microscope (JEOL Ltd., Tokyo, Japan). Hydrodynamic diameter of the particles (via dynamic light scattering) was measured using the Zetasizer NanoZS90 (Malvern Panalytical Ltd., Worcestershire, UK). Analysis was performed at a scattering angle of 90° and a temperature of 22 °C. All experiments were performed in triplicate, and the data are expressed as mean ± standard deviation (SD).

The fluorescence emission spectra characteristic to the colloidal AuNP-based samples were investigated using an FS5 spectrophotometer (Edinburgh Instruments, Livingston, UK). The spectrofluorometer is equipped with a 150 W Xe lamp light source with CW excitation in the UV–NIR range, Czerny–Turner monochromators for excitation and emission with a 1200 L/mm diffraction grating, and a PMT detector range of 185–980 nm. The samples were excited at 650 nm, and the spectra were acquired with 1 nm step size and a dwell time of 0.2 s.

The fluorescence emission of the samples based on AuFoN was investigated using a micro-Raman module (633 nm uRaman-Ci, Technospex, Singapore) integrated on a Nikon Ci-L upright microscope (Nikon Instruments, Tokyo, Japan). The samples were viewed and excited through a 4× (NA 0.13) Nikon Objective Lens (Nikon Instruments, Tokyo, Japan). Spectra were acquired with a 300 ms acquisition time. Additionally, polynomial smoothing of grade 3 was applied to each spectrum. The laser power was less than 1 mW at the sample. Several spectra were recorded from different areas on the sample, and the average spectrum together with the standard deviation was calculated for each sample and analysed. The reflectance spectra were acquired using the same system, which integrates a uSight 2100 module. The spectra were acquired using the same 4× microscope objective, 300 ms integration time, and 20 acquisitions. Grade-3 polynomial smoothing was applied to each spectrum. Several spectra were collected from various positions across the samples.

## 3. Results and Discussion

**Figure 1:**
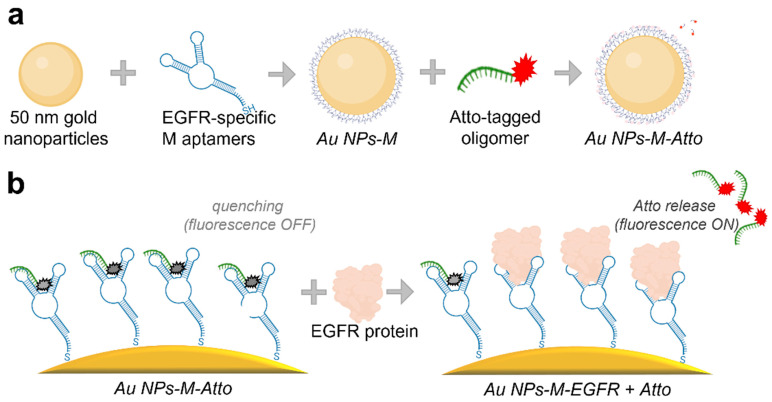
Schematics depicting (**a**) the functionalization steps for preparing the sensor (Protocol A), and (**b**) the surface-level molecular interactions for detection of EGFR.

The synthesized colloidal nanoparticles were functionalized using a stepwise protocol involving biomolecular components, aiming to create a competitive plasmonic sensing system, as schematized in Figure 1. Two types of DNA oligomers containing specific sequences and therefore specific functionalities were used: an EGFR-specific aptamer (M) chosen for its affinity toward the EGFR protein and a complementary DNA strand labelled with Atto647N fluorophore (Atto), used to monitor the binding–unbinding dynamics to/from the M aptamer via fluorescence.

The selected EGFR-specific aptamer was previously reported by Elligton et al. [44] to bind to EGFR with a dissociation constant, K_d_, of 2.4 nM [45], comparable to the binding affinity of therapeutic agents such as epidermal growth factor (EGF) or the mAb C225 [46]. This sequence, however, has been validated only in a few studies: for capturing circulating tumour cells [47], mediating intracellular delivery via endocytosis [48], supporting in vivo imaging through PET [49], and enhancing hollow gold nanosphere targeting and biodistribution in tumour models [50]. These applications underscore its potential as a robust molecular recognition element for EGFR-targeted biosensing.

The standard functionalization procedure, referred to as Protocol A, included the following steps: (i) aptamer immobilization by incubating the AuNPs with an excess of thiol group-containing aptamer for 1 h to ensure complete surface coverage via thiol–gold binding; (ii) first washing step—unbound aptamer was removed by centrifugation; the supernatant (SPN) containing unbound (excess) molecules was collected and measured, and the pellet (RES) was resuspended; (iii) functionalization with the fluorophore—the Atto-labelled complementary strand was added, allowing it to bind to the aptamer, thus bringing the Atto molecule into close proximity to the surface of the NPs; (iv) second washing step—unbound Atto-oligomer was removed by centrifugation; (v) incubation with EGFR protein for 1 h to test displacement of Atto-labelled strand via competitive binding; (vi) final washing step—a third centrifugation and removal of the supernatant containing the unbound and displaced molecules (the Atto oligomers detached from M in the presence of EGFR), yielding final sample for analysis. This protocol constitutes the core detection mechanism based on fluorescence signal modulation due to aptamer–EGFR interactions and is visually represented in Figure 1. Additionally, the Protocol A implied a reversed experimental design (Protocol A^REV^) to test the competitive binding in the case where the EGFR protein is introduced into the system before the Atto fluorophore-labelled strand. For this, the AuNPs were incubated with the M aptamer, followed by a washing and centrifuging step. Next, the resuspended NPs were incubated with the EGFR protein, followed by a second washing and centrifuging step. Finally, the NPs were functionalized with Atto, followed by a final washing step.

To evaluate the system reproducibility and robustness, Protocol A was repeated under identical conditions using a different AuNP colloidal batch. This repetition will be referred to as Protocol A′ and followed the same incubation and washing sequence. However, the fluorescence results showed marked deviations, suggesting instability and batch-dependent variability of the sensing platform, such as inconsistencies in the formation and properties of complexes made from AuNPs and the conjugating molecules. Therefore, to minimize colloidal instability and aggregation, two more protocols were designed which involved a reduced number of washing steps. Protocol B included the sequential incubation of AuNPs with the aptamer and the Atto-labelled strand, without intermediate washing. A single washing step was applied following fluorophore incubation. The resuspended NPs were then incubated with EGFR, followed by a final wash. A second strategy involving reduced washing steps was developed, Protocol C, which eliminated intermediate washing entirely to test whether the competitive binding mechanism could still function in a minimally handled system. For this, the AuNPs were sequentially incubated with the aptamer, fluorophore, and EGFR, and a single final washing step was performed after all components were introduced accordingly and were allowed to interact with the system. The entire experimental design is presented in Table 1.

### 3.1. Fluorescence Characterization of Atto in Solution

**Figure 2:**
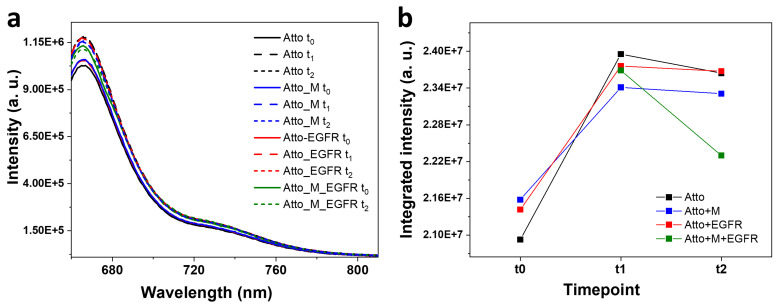
(**a**) Fluorescence emission spectra characteristic to Atto recorded at various time moments and conditions: in isolation, in the presence of the M aptamer, in the presence of the EGFR ligand, and in the presence of both the aptamer and the EGFR ligand. (**b**) The integrated intensity under the emission band calculated at each time moment: at t_0_, after 1 h incubation—t_1_, respectively 2 h after incubation—t_2_.

To establish a reference for subsequent nanoparticle-based experiments, an initial set of measurements was conducted to assess the fluorescence behaviour of the Atto-tagged oligomer in various biological environments. It was incubated under four conditions: (i) alone in solution, (ii) together with the M aptamer, (iii) together with the EGFR protein, and (iv) with both the aptamer and the protein. Fluorescence spectra were recorded at defined time intervals (t_0_ = 0 h, t_1_ = 1 h, t_2_ = 2 h) and are represented in Figure 2. Quantum yield (QY) measurements were also performed to assess the photophysical behaviour of Atto in each context. At t_0_, Atto exhibited a well-defined emission spectrum, serving as a baseline for subsequent comparisons. Upon mixing Atto with the M aptamer, the initial fluorescence intensity increased slightly (3%) compared to Atto alone. This minor enhancement may result from structural interactions or local environmental changes introduced by the aptamer. After the first hour of incubation, the free Atto displayed a fluorescence increase of 14% relative to its t_0_ value. The Atto–aptamer mixture increased by 8.4% compared to its own initial signal. However, when comparing the two systems at t_1_, the Atto–aptamer system showed 2.2% lower fluorescence intensity compared to the free Atto. This small reduction suggests that, although the aptamer may provide some stabilization, it also introduces minor quenching effects.

Subsequently, the system containing Atto and the aptamer was incubated with the EGFR ligand. At t_0_ (immediately after EGFR addition, but after 1 h incubation with the M aptamer), the fluorescence intensity decreased by 1.1% relative to the free Atto at t_1_, and increased by 1.17% relative to the Atto–aptamer system at t_1_. After another hour of incubation with EGFR (t_2_), the system showed 4% intensity decrease compared to the system composed of Atto and the aptamer at t_2_. This suggests that the initial fluorescence boost may result from transient structural rearrangements or short-lived interactions. This subtle decay could reflect conformational changes in the aptamer–fluorophore complex upon EGFR binding or direct interactions between EGFR and Atto, altering its emission properties. When Atto was incubated only with EGFR, the initial fluorescence at t_0_ revealed a modest increase compared to Atto alone at t_0_. After 1 h, the fluorescence intensity decreased relative to the free Atto, although it remained slightly higher than the one corresponding to the Atto–aptamer mixture. These results indicate that EGFR alone has a limited effect on Atto fluorescence and that its influence is weaker and less structured than that of the aptamer or the combined system.

The QYs were measured for all four systems. The highest value was obtained for Atto (34%), followed by Atto incubated with both the aptamer and EGFR (31%), Atto incubated only with the aptamer (29%), and Atto incubated with EGFR only (25%). The QY of Atto in aqueous media should be higher, around 65%, as given by the producer; however, when bound to a DNA oligomer and given the aqueous media in which the measurements were performed, additional non-radiative decay pathways can be introduced, leading to a reduction in QY. It was shown that dye–DNA interaction restricts the mobility of the dye, which increases the anisotropy, but may reduce radiative decay rates and implicitly lead to a drop in QY [51]. These results suggest that the aptamer exerts a subtle but measurable effect on the photophysical properties of Atto, such as a slight stabilization and minor quenching effects. The EGFR protein alone decreases the QY, likely due to environmental interactions or reorganization of the fluorophore’s immediate surroundings. The system containing all three components maintains a relatively high QY (31%), suggesting an altered dye environment that diminishes some quenching pathways.

### 3.2. Competitive Binding and Fluorescence Quenching: Early Evaluation of AuNPs-Aptamer Sensors

**Figure 3:**
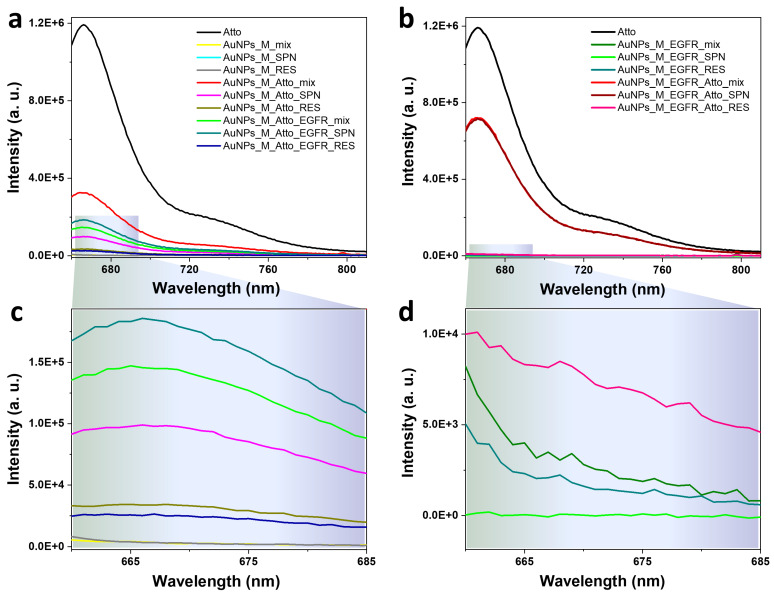
Fluorescence emission spectra characteristic to the colloidal gold nanoparticles functionalized by (**a**,**c**) Protocol A: direct competitive binding using AuNPs incubated with the aptamer, the fluorophore, and the protein, and by (**b**,**d**) Protocol A^REV^: the reversed assay based on functionalizing the AuNPs with the EGFR protein, followed by the addition of the Atto fluorophore. Spectra were recorded after each incubation and washing step. Panels (**c**,**d**) show zoom-in details of the low-intensity spectra given in panels (**a**,**b**).

Initial characterization of the Protocol A system based on AuNPs was conducted to evaluate their potential as optical biosensing platforms for protein detection in solution. Fluorescence emission was monitored to determine the binding and displacement dynamics between the fluorophore and the target protein; the spectra are shown in Figure 3a,c. The applied protocol for incubation of the AuNPs was as described in Figure 1 and Table 1. No detectable fluorescence was observed for the AuNPs functionalized only with the aptamer (Figure 3a), which is in agreement with the absence of non-specific Atto emission. Upon incubation with Atto, a significant decrease (73%) in the characteristic fluorescence was observed relative to that of free Atto, indicating the quenching of the Atto signal as it interacted with the AuNP surfaces and that energy was transferred non-radiatively due to its proximity to the metal surface. Additionally, a small percentage of the fluorescence decrease could also be induced by the complex between Atto and the aptamer, as seen from Figure 2. Subsequent washing steps yielded two fractions: the supernatant (SPN) and the resuspended nanoparticles (RES). The SPN sample retained 69% lower intensity compared to the aptamer-Atto mix, while the RES sample showed an even more pronounced 89% decrease. The higher fluorescence in the SPN fraction suggests the presence of Atto molecules unbound or weakly associated with the AuNPs–aptamer complex that were freed during the centrifugation process, whereas the signal suppression in the RES fraction confirms that a fraction of the incubated Atto molecules remain in close contact with the nanoparticle surface, where quenching persists.

Next, EGFR was introduced into the AuNPs-aptamer-Atto mixture to test displacement. The resulting mix sample showed a 318% increase in fluorescence relative to the RES control. This strongly supports the hypothesis that EGFR preferentially binds the aptamer, displacing the Atto-labelled complementary strand and thereby restoring fluorescence. This displacement confirms the target specificity of the sensor and provides a clear optical readout. Subsequent washing of the EGFR-incubated mix again generated SPN and RES samples. The SPN exhibited a 20% fluorescence increase compared to the unwashed mix, consistent with Atto being released into the supernatant post-displacement. Conversely, the RES fraction showed an 81% decrease, suggesting some residual Atto remains bound to the AuNPs and continues to experience quenching.

To further confirm competitive binding specificity, the reversed assay (Protocol A^REV^) was conducted in which AuNPs functionalized with the aptamer were first incubated with EGFR, followed by addition of the Atto fluorophore. The resulting spectra (Figure 3b,d) showed that the fluorescence intensity of the mix sample (aptamer + EGFR + Atto) was equivalent to that of its corresponding SPN, indicating that Atto could not bind to the NPs once EGFR was pre-bound to the aptamer. This demonstrates that EGFR blocks the binding sites for the complementary Atto strands and prevents its association with the AuNPs–aptamer complex.

**Figure 4:**
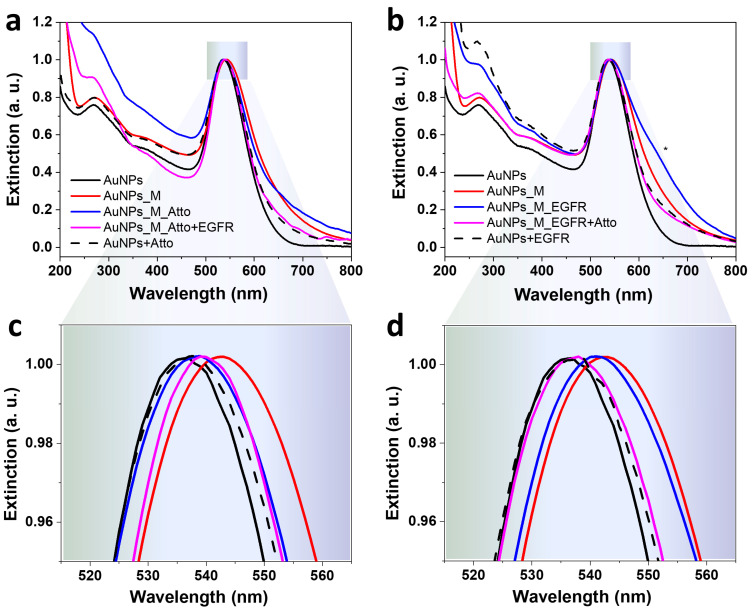
UV–Vis extinction spectra characteristic to the colloidal gold nanoparticles functionalized by (**a**,**c**) Protocol A: direct competitive binding using AuNPs incubated with the aptamer, the fluorophore, and the protein; and by (**b**,**d**) Protocol A^REV^: the reversed assay based on functionalizing the AuNPs with the aptamer, the EGFR protein, followed by the addition of Atto. The * symbol marks the effect of the formation of NP aggregates. Panels (**c**,**d**) show zoom-in details of the spectra in panels (**a**,**b**).

To verify the efficiency of the oligomer functionalization, the optical response of the AuNP samples at various stages was investigated using UV–Vis absorption spectroscopy, and the results are given in Figure 4. The synthesized nanoparticles present an LSPR maximum at 537 nm, which corresponds to spherical nanoparticles of about 50 nm, a morphological feature also confirmed by TEM images and DLS data in Supplementary Figure S1 and Table S1. The incubation of the AuNPs with the aptamer induces a 6 nm red-shift of the LSPR maximum, confirming the presence of the aptamer on the metallic surface. The introduction of Atto into the system leads to a blue-shift, while the additional incubation with the EGFR protein presents a weak red-shift relative to the AuNPs–aptamer–Atto fluorophore complex. Moreover, the sequential aptamer and fluorophore incubation induces a slight broadening of the full width half maximum (FWHM) of the LSPR band, which indicates the potential formation of AuNPs clusters in the colloidal solution. This in-liquid assembly of the particles can also lead to stronger fluorescence signals due to the hot spots created by the aggregated NPs [52]. The systems obtained using the reversed assay, Protocol A^REV^, present similar outcomes. The incubation with EGFR of the AuNPs–aptamer complex induces a blue-shift of the LSPR relative to the position of the maximum detected for the AuNPs–aptamer system. A further blue-shifting is Induced by the incubation with Atto, as well. Both the aptamer and the protein incubation generate the formation of small NP aggregates, marked with an * in Figure 4b. UV–Vis extinction spectra of AuNPs have a characteristic peak at 270 nm that appears due to interband absorption, an electronic transition which involves electron–hole pair recombination within the metal, rather than the collective oscillation of electrons on the nanoparticle surface responsible for SPR. Coincidently, DNA and aptamers have a characteristic absorption at 260 nm because the aromatic rings of the purine and pyrimidine bases in nucleic acids and proteins have a specific absorption at 280 nm due to the presence of aromatic amino acids (tryptophan, tyrosine, and histidine) [53]. Depending on the concentration of aptamer or protein, respectively, some absorption should be observable in the spectra. However, the concentrations measured herein were below such a limit, with the exception of the AuNPs + EGFR mixture (dotted line in Figure 4b). It is worth noting that the spectra presented in Figure 4 are normalised to unity at the LSPR peak, so the more washing steps the particles underwent, the more aggregation/clustering occurred, and thus the more the aspect of the UV region was over-enhanced.

These initial fluorescence-based experiments provide compelling evidence that the AuNPs–aptamer platform effectively discriminates between the complementary DNA strand and the target protein via competitive binding. The system exhibited strong quenching behaviour consistent with close-range fluorophore–nanoparticle interaction and demonstrated reliable signal restoration upon displacement.

### 3.3. System Stability Testing

In a subsequent set of experiments aiming to assess the reproducibility of the fluorescence-based detection system described in Protocol A, the previously shown stepwise procedure was repeated on a different day and using a different batch of colloidal AuNPs. Although the employed chemical synthesis method is widely used in research experiments and is known for yielding stable, reproducible colloidal batches, slight differences can still be observed by UV–Vis spectroscopy. The UV–Vis spectra characteristic to each batch are given in Supplementary Figure S2. As can be seen, the peak position of the LSPR shifts only 1–2 nm across batches. This is a very small value, within the normal synthesis variability. However, a FWHM broadening was observed, especially towards the NIR spectral range. Despite this broadening being small, it can suggest increased polydispersity and/or onset of aggregation, due to altered surface chemistry. These changes can modify the near-field distribution and surface functionalization outcome.

**Figure 5:**
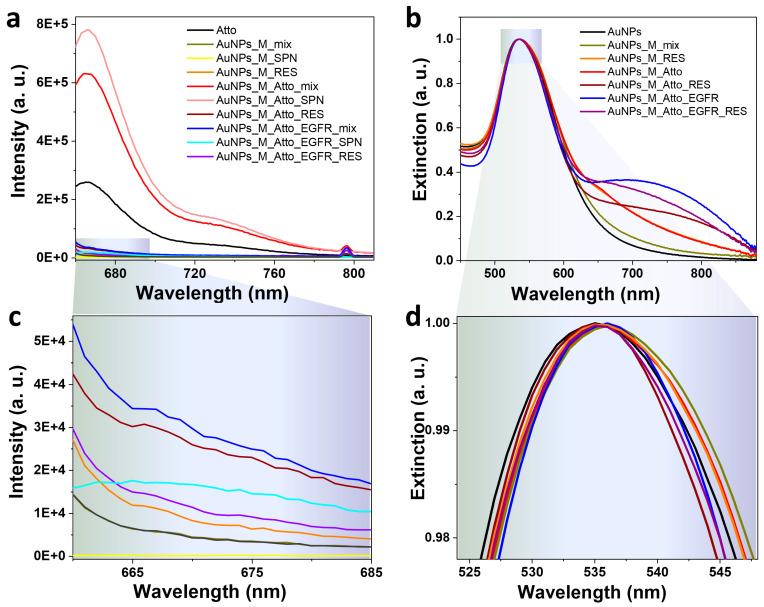
(**a**,**c**) Fluorescence emission spectra and (**b**,**d**) UV–Vis extinction spectra characteristic to the AuNP-based system obtained using Protocol A′ (repeated incubation and washing protocol using a new batch of colloidal NPs). Panels (**c**,**d**) show zoom-in details of the spectra in panels (**a**,**b**).

This second experimental run, referred to as Protocol A′, followed the identical stepwise incubation and washing approach: incubation with aptamer, Atto, and EGFR, with washing and centrifugation after each step. This allowed a direct comparison to the previous results. However, as presented in Figure 5a,c, the fluorescence data obtained from Protocol A′ exhibited significantly different behaviour compared to the results from Protocol A. The mix sample obtained after incubating the AuNPs with the aptamer and the fluorophore showed a 142% increase in fluorescence intensity compared to Atto alone. This enhancement could suggest plasmon-enhanced fluorescence, possibly due to reduced non-radiative deactivation pathways when the fluorophore interacts with the aptamer-functionalized AuNPs. A possible explanation for the large modification observed in the fluorescence signal compared to Protocol A can be related to small changes in spacer length or aptamer conformation, which can flip a dye from the quenching to enhancement regime, as fluorescence enhancement is extremely distance-dependent [54]. However, after centrifugation, the SPN sample exhibited a further 23% increase in fluorescence compared to the mix sample, likely due to the free Atto molecules in solution. In contrast, the RES sample presented a drastic 93.7% decrease in intensity, suggesting that very few Atto molecules remained attached to the AuNP surfaces. Additionally, considering the results obtained from Protocol A (see Figure 3), fluorophore molecules experience fluorescence quenching in the vicinity of the metallic surface, a result contrasting with the currently discussed data. These results may indicate that, in this case, the binding between the fluorophore and the AuNPs–M complex is very weak.

Unexpectedly, incubation with the EGFR protein resulted in a collapse of the fluorescence signal. Neither the mix nor the RES sample displayed a clear Atto emission peak at 666 nm. Only the SPN sample retained a measurable signal, implying that the Atto molecules were fully displaced by the EGFR protein and appeared in the supernatant. The complete absence of emission in the resuspended particles indicates that any fluorophores remaining bound to the AuNPs were either displaced or completely quenched. This result contrasts sharply with earlier experiments, where a distinct increase in fluorescence was observed in the mix sample after EGFR addition, supporting the competitive binding hypothesis. On the contrary, the Protocol A′ system gave an inconsistent response, and the expected signal trends were not reproduced even though the experimental procedure was unchanged.

The UV–Vis extinction spectra shown in Figure 5b,d further illuminate the system’s instability. All spectra were normalized to highlight subtle shifts in the LSPR peak and associated aggregation behaviour. Initial functionalization with the M aptamer led to a 2 nm red-shift, consistent with changes in the local refractive index upon aptamer binding. However, following centrifugation and resuspension, this shift was lost, possibly indicating removal of weakly bound aptamer. Further, an increase in extinction in the 650–850 nm spectral range was observed, especially in the RES fractions after Atto and EGFR incubations. This spectral feature indicates significant AuNPs aggregation, which may stem from disrupted colloidal stability during washing steps or from interparticle crosslinking facilitated by aptamer/protein interactions. The distorted LSPR band shape and the extended red/NIR tail are consistent with both interparticle plasmonic coupling and increased fluorophore–surface interactions, both of which may contribute to the observed signal loss. These results emphasize a critical limitation: despite following an identical protocol, the system failed to reproduce the signal modulation seen in earlier measurements. This suggests that NP surface conditions, colloidal stability, or batch-to-batch variability may have a stronger influence than initially anticipated. The significant fluorescence intensity in the mix and SPN samples, coupled with the near-total quenching in the RES fraction, points to complex dynamics involving fluorophore binding affinity, surface accessibility, and aggregation state. The absence of a signal increase following EGFR incubation raises questions about aptamer integrity and target binding efficiency, both of which may degrade over time or vary between NPs batches. Importantly, UV–Vis–NIR data confirm that aggregation is an issue that could mask or distort optical signals. These observations call for improved surface passivation and further exploration of one-pot conjugation strategies to reduce variability. Ultimately, while the original sensing concept remains promising, these later results highlight the challenges of reproducibility and surface chemistry control in nanoparticle-based biosensing systems. Washing protocols, ligand desorption, and inadequate spacers are well-recognized causes of irreproducibility in plasmonic biosensing, as chemical functionalization of NP surfaces comes with practical challenges [55]. Small changes in washing and post-functionalization handling can alter the packing density and thickness of the M aptamer layer on the surface of AuNPs, thereby influencing whether the observed fluorescence emission signal will be quenched or enhanced.

### 3.4. Improved Fluorescence Response Through Reduced Washing: Protocols B and C

In order to reduce the NPs aggregation resulting from washing and centrifuging the samples and the signal loss observed in earlier trials, a modified experimental design was implemented that minimized or eliminated washing steps. Centrifugation and repeated resuspension are known to destabilize colloidal systems and partially desorb aptamers, resulting in variable fluorescence responses [56]. By reducing perturbations to the AuNPs–aptamer system, these simplified protocols aimed to preserve colloidal stability and maintain the integrity of the recognition layer, in accordance with previous reports identifying aptamer immobilization chemistry, surface passivation, and handling procedures as critical sources of variability in aptasensor development [56] Two variations of Protocol A were introduced: (i) Protocol B—involving one intermediate washing step after the AuNPs were incubated sequentially with the aptamer and Atto, followed by the incubation with the EGFR protein of the resuspended NPs; and (ii) Protocol C—which implied eliminating intermediate washes entirely and performing a single final washing and centrifugation step only after the final incubation of the NPs with EGFR.

**Figure 6:**
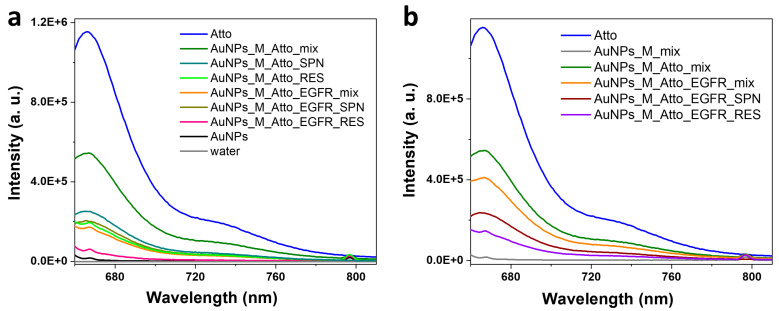
Fluorescence spectra characteristic to the AuNP-based systems obtained by (**a**) Protocol B, implying one intermediate washing step; (**b**) Protocol C, based on a single final wash of the AuNPs.

Figure 6a presents the fluorescence spectra characteristic to the system obtained using Protocol B. When Atto was added to aptamer-functionalized AuNPs, the fluorescence decreased as expected, due to quenching by the AuNPs. The fluorescence signal corresponding to the RES sample showed an over 50% decrease compared to Atto alone, indicating removal of the free or weakly bound fluorophore molecules. Following EGFR incubation and a final wash, the fluorescence dropped by another 36%. The RES sample showed minimal emission, while the SPN sample showed a 22% increase in intensity compared to the unwashed mix, suggesting that Atto was partially displaced by the EGFR and remained unquenched in the supernatant. For the system obtained using Protocol C, which involved only a final washing step, the fluorescence spectra are presented in Figure 6b. It can be observed that the trend detected for Protocol B persisted in this case, as well. The initial fluorescence of the AuNPs–aptamer–Atto system was strong, but decreased by 25% after EGFR protein incubation. The RES sample presented a further slightly diminished signal, while the SPN sample exhibited an increase in intensity compared to the corresponding RES sample, indicating that even in this minimally handled system, EGFR displaces Atto, leading to signal redistribution and potential detectability in the supernatant.

To further probe the system’s photophysical behaviour, QY measurements were performed. In the case of the single wash system, Protocol C, the mix sample from the unwashed AuNPs–aptamer–Atto system showed a very low QY (~0.75%), despite a measurable fluorescence, possibly indicating a balance between emission from unbound Atto and quenching near AuNPs. Upon EGFR addition, the QY dropped further, to 0.34%, consistent with increased quenching or reorganization within the system. Notably, the QY of the corresponding SPN sample jumped to 5.9%, highlighting efficient emission from freed Atto molecules. For the intermediate-wash system, Protocol B, the QY of the SPN sample post-EGFR incubation was slightly lower (2.4%), likely due to earlier removal of excess Atto during the wash step, leaving behind primarily the bound fraction. These results underscore the importance of balancing washing steps for specificity against preserving overall system stability.

**Figure 7:**
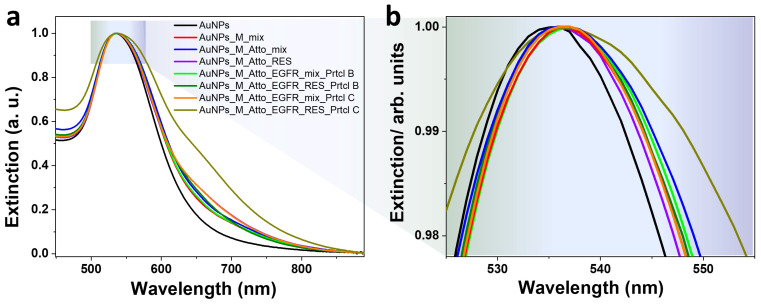
(**a**) UV–Vis extinction spectra characteristic to the AuNP-based systems obtained using Protocol B and Protocol C. (**b**) The panel presents a zoom-in detail of the spectra.

Figure 7 presents the UV–Vis–NIR extinction spectra corresponding to the AuNPs obtained using Protocols B and C. Unlike in previous trials, AuNP aggregation was significantly reduced in the systems where intermediate washing was eliminated or minimized. The strongest aggregation was observed in the intermediate-wash configuration or Protocol B, specifically for the resuspended AuNPs obtained after all incubation steps and the final washing procedure, where visible tailing in the red/NIR region and LSPR broadening indicates extensive interparticle coupling. In all systems, the aptamer-induced LSPR red-shift (~2 nm) was preserved, suggesting that surface functionalization still occurred efficiently even in the absence of repeated washing. This shift confirms successful aptamer binding and a stable local refractive index change, crucial for sensing applications. However, due to the rather small shift compared to the initially presented results which showed a 6 nm red-shift, weak binding of the aptamer to the surface of the AuNPs could be implied.

This final experimental series demonstrates that removing intermediate washing steps enhances system performance by reducing AuNPs aggregation, as shown by UV–Vis extinction profiles, preserving fluorescence and avoiding signal loss due to particle instability, as well as enabling consistent detection of EGFR-induced fluorophore displacement, confirmed by increased SPN fluorescence and QY.

### 3.5. AuFoN Solid Substrate-Based Sensing

**Figure 8:**
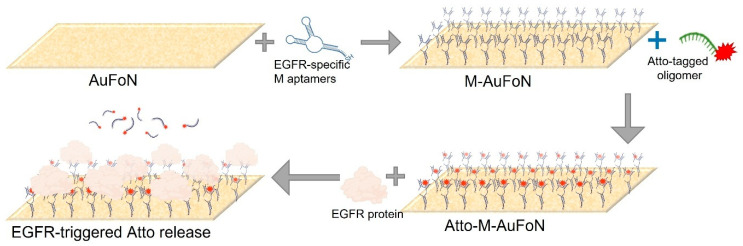
Schematics depicting the functionalization of the AuFoN nanostructured gold solid substrates.

Given the reproducibility challenges and signal instability encountered with colloidal AuNPs, the study was extended to investigate plasmonic nanostructured substrates. Specifically, AuFoN was employed as an alternative sensing surface. These platforms offer higher stability and allow more controlled surface chemistry and repeated handling. Additionally, substrates are selected for biosensing due to their unique optical properties, including LSPR and increased electromagnetic fields, which can contribute to fluorescence signal enhancement [57]. Their large surface area and tunable morphology makes them promising candidates for biosensing applications [14,58]. The experimental procedure was maintained to allow direct comparison with the colloidal AuNPs; therefore, the same functionalization sequence was applied: successive incubation with the M aptamer, the Atto fluorophore-labelled complementary strand, and the EGFR protein, with washing steps after each stage. A schematic of the surface modification workflow is shown in Figure 8.

**Figure 9:**
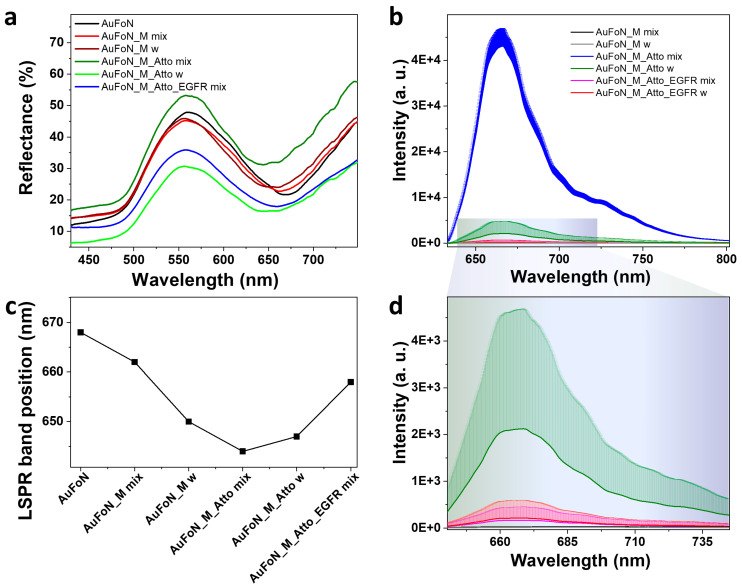
(**a**) Reflectance and (**b**) fluorescence emission spectra acquired from the AuFoN incubated with the same protocol as the one used for colloidal nanoparticles. (**c**) The shifting of the LSPR minimum for each investigated sample. (**d**) A close-up of the fluorescence emission spectra shown in (**b**). The letter w in the legends refers to washing step.

First, the AuFoN substrate was investigated using reflectance spectroscopy following each incubation step, and the spectra are given in Figure 9a. The plasmon resonance in the AuFoN is indicated by the reflectance minimum observed here in the 650–660 nm range and shown in Figure 9c. Note that the optical response of the AuFoN system can be broadly adjusted through the nanosphere size and gold film thickness [59]. The polystyrene nanosphere diameter of 500 nm and gold film thickness of 120 nm were selected to obtain the plasmon resonance in the 600–700 nm spectral range, matching the fluorophore emission. Interestingly, the AuFoN substrate exhibits a blue-shift of the LSPR minimum upon incubation with biomolecular layers, contrarily to the AuNPs, which, typically, present a red-shifted absorption maximum. Previously, negative surface plasmon resonance signals were reported when the ligand immobilized on the surface underwent a significant conformational transition upon analyte recognition, such as folding or stretching [60]. Negative refractive index increment deviations were observed both in solutions and on substrates for protein binding and aptamer folding. The blue-shift was assigned to a negative complex (ligand/analyte) refractive index increment (RII) deviation from the sum of the RII of the individual entities [61]. Therefore, the observed shift in our spectra after aptamer incubation confirmed its immobilization onto the surface. The functionalization with Atto presented an additional LSPR shift indicating that the dye-labelled oligomer was successfully added to the system. The incubation with the EGFR induced a slight red-shift of the plasmon band compared to the Atto incubation, confirming interaction or surface proximity. These modifications indicate the biochemical functionalization was induced as expected.

Strong fluorescence was obtained immediately after Atto immobilization (Figure 9b). Substrate washing caused a pronounced decrease in fluorescence, indicating that most of the fluorophore molecules were either loosely attached or experienced strong quenching when brought close to the metal surface. The fluorescence intensity did not recover post-EGFR incubation. On the contrary, it showed an additional decrease, implying that washing effectively removed most non-specifically bound fluorophores and that EGFR could not displace the strongly attached ones. The final washing step yielded a fluorescence signal nearly identical to that corresponding to EGFR incubation, suggesting that no additional Atto displacement or structural rearrangement was induced by the EGFR functionalization. Therefore, the fluorescence loss indicates that, under the current conditions, most Atto molecules are not stably retained on the nanostructured gold surface and that the drop in fluorescence signal is due to washing and not necessarily to EGFR-induced competitive binding. Despite the reflectance spectra confirming molecular adsorption, stable fluorophore retention is not guaranteed, as the fluorescence measurements suggest that the Atto-labelled strand is only weakly retained, and it is easily removed by washing.

We emphasize that the current study was intended as a mechanistic investigation of the role of fluorophore–aptamer–AuNP interactions, of the interplay between variations among batches of colloidal AuNPs, and of how surface chemistry and handling protocols (washing, centrifuging) influence plasmon-enhanced fluorescence performance, rather than an effort to deliver a fully optimized biosensor. The key limitations of the platform developed here include (i) sensitivity to variations between different colloidal AuNPs batches, which alters the near-field distribution and the balance between fluorescence enhancement and quenching, and (ii) loss of signal following repeated washing steps due to partial detachment induced by the surface chemistry differences between the used colloidal AuNPs batches. The presented variable outcomes identify as some of the most important issues that must be addressed when designing aptamer-plasmon-based biosensors.

A critical limitation of many plasmonic aptasensors is related to the poor reproducibility linked to surface chemistry, colloid variability, and post-functionalization handling. Reproducibility issues associated with immobilization chemistry [55] or small changes in dye–metal distance [54] are documented in the literature. For example, surface passivation with thiol-terminated polyethylene glycol or oligo spacers can reduce non-specific adsorption and stabilize colloids, thereby improving reproducibility [62]. In practice, chemical adsorption and covalent binding are frequently used to immobilize biorecognition elements. Among these, self-assembled monolayer (SAM) remains one of the most widely used strategies, enabling stable immobilization of proteins, oligonucleotides, and peptide nucleic acids [56]. Thiol-gold chemistry is particularly common, as thiol groups have strong affinity for gold surfaces, while the alkyl backbone secures the biomolecule to the substrate. However, it has been reported that thiolated aptamers do not exclusively bind to gold surfaces via the Au-S bond, as nonspecific interactions via nitrogen atoms can occur, potentially restricting target recognition [63]. Strategies such as the addition of mercaptohexanol have been employed to displace non-specifically adsorbed aptamers and promote upright orientation [56], while alkanethiols of different lengths have been shown to optimize aptamer folding and target binding. Despite these advances, both approaches still face the major challenge of biofouling, which negatively affects the sensitivity, reproducibility, stability, and reliability of biosensors [64]. Additionally, reproducibility is often compromised by batch-to-batch variations in NPs size, shape, and surface charge, which directly affect immobilization efficiency and dye–metal coupling [65].

## 4. Conclusions

This study compared two types of plasmonic sensing platforms, colloidal AuNPs suspensions and film-based solid substrates, namely gold film over nanospheres, functionalized with an EGFR-specific aptamer and a fluorophore-labelled DNA oligomer. Although both systems were designed to exploit plasmon-enhanced fluorescence for biomarker detection, their performance was significantly different depending on architecture and handling protocol. The colloidal AuNPs-based system showed potential for fluorescence modulation through competitive binding. Upon incubation with Atto, effective quenching of the fluorophore’s signal as it interacted with the AuNP surface and non-radiative energy transfer were observed. The incubation with EGFR revealed that the biomarker preferentially binds the aptamer, displacing Atto and thereby restoring fluorescence. The competitive binding specificity was confirmed by incubating the aptamer-functionalized AuNPs with EGFR, followed by addition of Atto. The results revealed that Atto could not bind to the NPs once EGFR was pre-bound to the aptamer. Additional investigations revealed that the AuNPs were sensitive to the applied protocol involving washing steps, colloidal stability, and batch-to-batch variability and that complex dynamics are involved in fluorophore binding affinity, surface accessibility, and aggregation state. It was shown that multiple washing and centrifugation steps led to colloid aggregation and quenched fluorescence, while reducing these steps improved signal retention and enabled detection of fluorophore displacement by EGFR in the supernatant. In contrast, the film-based systems demonstrated poor retention of Atto after washing. A strong fluorescence signal was acquired after Atto immobilization; however, the AuFoN did not retain the Atto–M complex after washing. Indistinguishable EGFR-induced displacement from non-specific losses was obtained. Future optimizations are needed, such as surface functionalization of the substrates that can enhance binding specificity, test alternative linkers, and optimize aptamer/fluorophore concentrations. Although for the AuFoN, we did not achieve the specific recognition effect as initially hypothesized, these findings underscore the inherent challenges in aptamer-based plasmonic biosensing and provide a roadmap for future improvements. While colloidal AuNPs offer tunable behaviour and potential for biomolecular interaction monitoring, they are highly sensitive to experimental conditions such as washing. Under current conditions, film substrates provide high signal amplification initially, but lack retention and displacement specificity, likely due to suboptimal surface binding strategies. These results emphasize the critical importance of surface chemistry, aptamer–fluorophore affinity, and sample handling in biosensor design.

## Figures and Tables

**Table 1:** Summary of the experimental designs/protocols for functionalization of the colloidal AuNPs used in this study.

Protocol	Experimental Design
Protocol A and A′	Functionalization with the M aptamer—washing and centrifuging Functionalization with Atto—washing and centrifuging Functionalization with the EGFR biomarker—washing and centrifuging
Protocol A^REV^	Functionalization with the M aptamer—washing and centrifuging Functionalization with the EGFR biomarker—washing and centrifuging Functionalization with Atto—washing and centrifuging
Protocol B	Functionalization with the M aptamer Functionalization with Atto—washing and centrifuging Functionalization with the EGFR biomarker—washing and centrifuging
Protocol C	Functionalization with the M Functionalization with Atto Functionalization with the EGFR biomarker—washing and centrifuging

## Data Availability

The data that support the findings of this study are available at https://doi.org/10.6084/m9.figshare.30355231.v1.

## Supplementary Materials

The following supporting information can be downloaded at: https://www.mdpi.com/article/10.3390/bios15100699/s1, Figure S1: TEM images of colloidal gold nanoparticles (AuNPs) at various magnifications. Table S1: The hydrodynamic diameters and polydispersity indexes (PDI) of gold nanoparticles (AuNPs) in different stages of functionalization. Figure S2: UV-Vis extinction spectra of colloidal AuNPs from different batches.
