# Supplementary material for: Plasmonic Nanosensors for EGFR Detection: Optimizing Aptamer-Based Competitive Displacement Assays
Source: Biosensors (Basel). 2025 Oct 15;15(10):699. doi: 10.3390/bios15100699 (PMC12564422; doi:10.3390/bios15100699)
Supplement: Supplementary file 1 [file biosensors-15-00699-s001.zip › biosensors-3871789-supplementary.pdf]

Supplementary Material

Plasmonic Nanosensors for EGFR Detection: Optimizing Aptamer-Based Competitive Displacement Assays

Alexandra Falamas [1,†], Andra-Sorina Tatar [1,†], Sanda Boca [1,2,‡] and Cosmin Farcău [1,*]

[1]National Institute for Research and Development of Isotopic and Molecular Technologies, 67-103 Donat, 400293 Cluj-Napoca, Romania

[2] Interdisciplinary Research Institute in Bio-Nano-Sciences, Babes-Bolyai University, 42 Treboniu Laurian, 400271 Cluj-Napoca, Romania

Corresponding author: cfarcau@itim-cj.ro

† These authors contributed equally to this work.

‡ For this author the primary affiliation is 2.

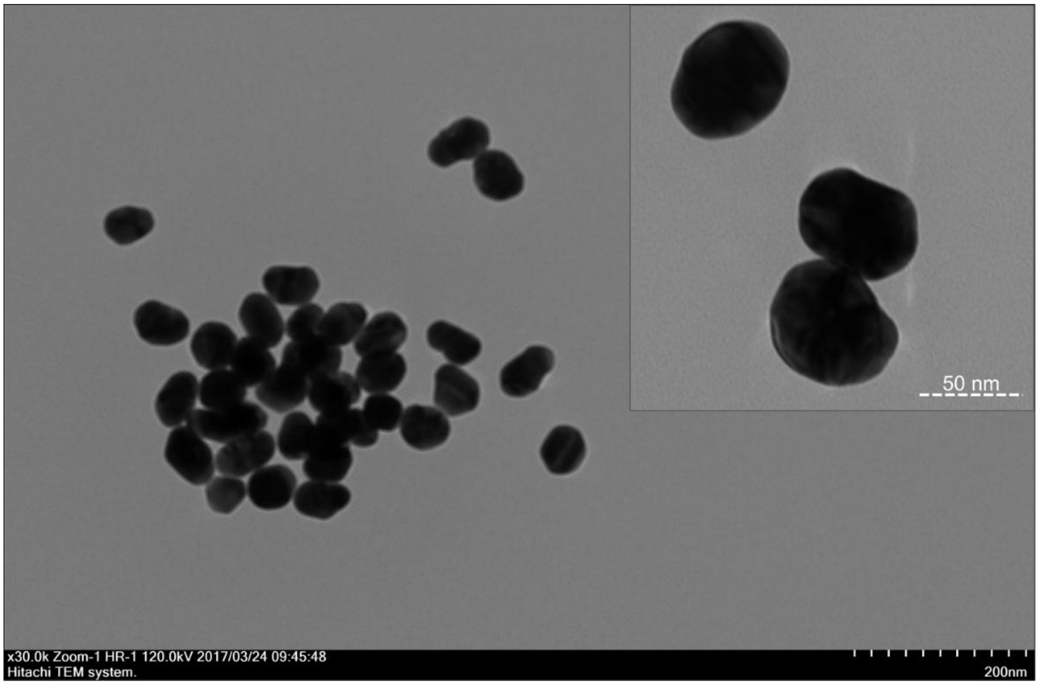

Figure S1. TEM images of colloidal gold nanoparticles (AuNPs) at various magnifications.

Table S1. The hydrodynamic diameters and polydispersity indexes (PDI) of gold nanoparticles (AuNPs) in different stages of functionalization.

SAMPLE NAME	HYDRODYNAMIC DIAMETER (NM)	PDI
AUNPS	72.78 ±0.72	0.561
ATTO	152.2 ±3.73	0.192
AUNPS_M	85.08 ±2.4	0.550
AUNPS_M_ATTO_MIX	85.02 ±4.1	0.515
AUNPS_M_ATTO	83.33 ±4.6	0.499
AUNPS_M_ATTO_EGFR	83.11±1.03	0.488

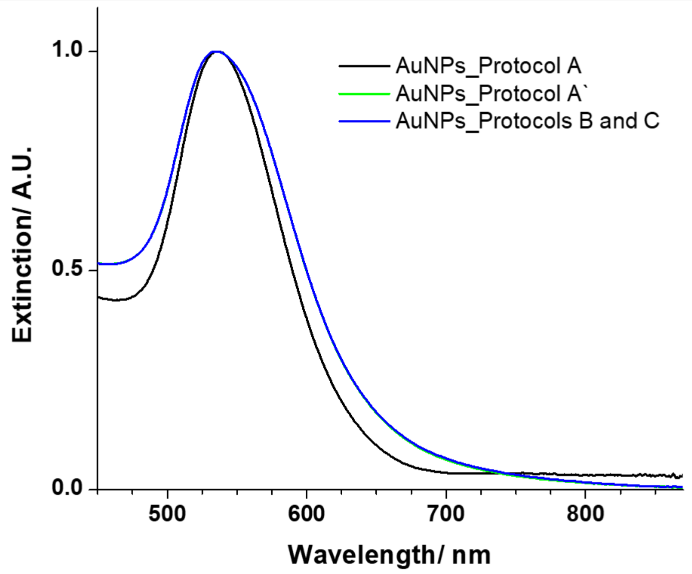

Figure S2. UV-Vis extinction spectra of colloidal AuNPs from different batches.
